# Dr. Darwin Colvin

**Published:** 1911-03

**Authors:** 


Dr. Darwin Colvin, of Clyde, N. Y., died at his home January 8, 1911, in the eighty-ninth year of his age. He graduated from Geneva Medical College in 1844. He served as a surgeon of volunteers during the Civil War, was one of the founders of the New York State Medical Association and its president in 1896.

